# An autism-associated calcium channel variant causes defects in neuronal polarity in the ALM neuron of *C. elegans*

**DOI:** 10.17912/micropub.biology.000378

**Published:** 2021-04-01

**Authors:** Tyler Buddell, Christopher C Quinn

**Affiliations:** 1 University of Wisconsin, Milwaukee, WI USA

## Abstract

Variants of the *CACNA1C *voltage-gated calcium channel gene have been associated with autism and other neurodevelopmental disorders including bipolar disorder, schizophrenia, and ADHD. The Timothy syndrome mutation is a rare *de novo* gain-of-function variant in *CACNA1C* that causes autism with high penetrance, providing a powerful avenue into investigating the role of *CACNA1C* variants in neurodevelopmental disorders. In our previous work, we demonstrated that an *egl-19(gof)* mutation, which is equivalent to the Timothy syndrome mutation in *CACNA1C,* can disrupt termination of the PLM axon in *C. elegans*. Here, we report a novel phenotype for the *egl-19(gof)* mutation, whereby it causes the growth of an ectopic process from the ALM cell body. We also extend our previous results to show that the *egl-19(gof)* mutation causes axon termination defects not only in the PLM axon, but also in the ALM axon. These results suggest that the Timothy syndrome mutation can disrupt multiple steps of axon development. Further work exploring the molecular mechanisms that underlie these perturbations in neuronal polarity and axon termination will give us better understanding of how variants in *CACNA1C* contribute to the axonal defects that underlie autism.

**Figure 1. The egl-19(gof) Timothy syndrome mutation causes defects in axon termination and neuronal polarity:**
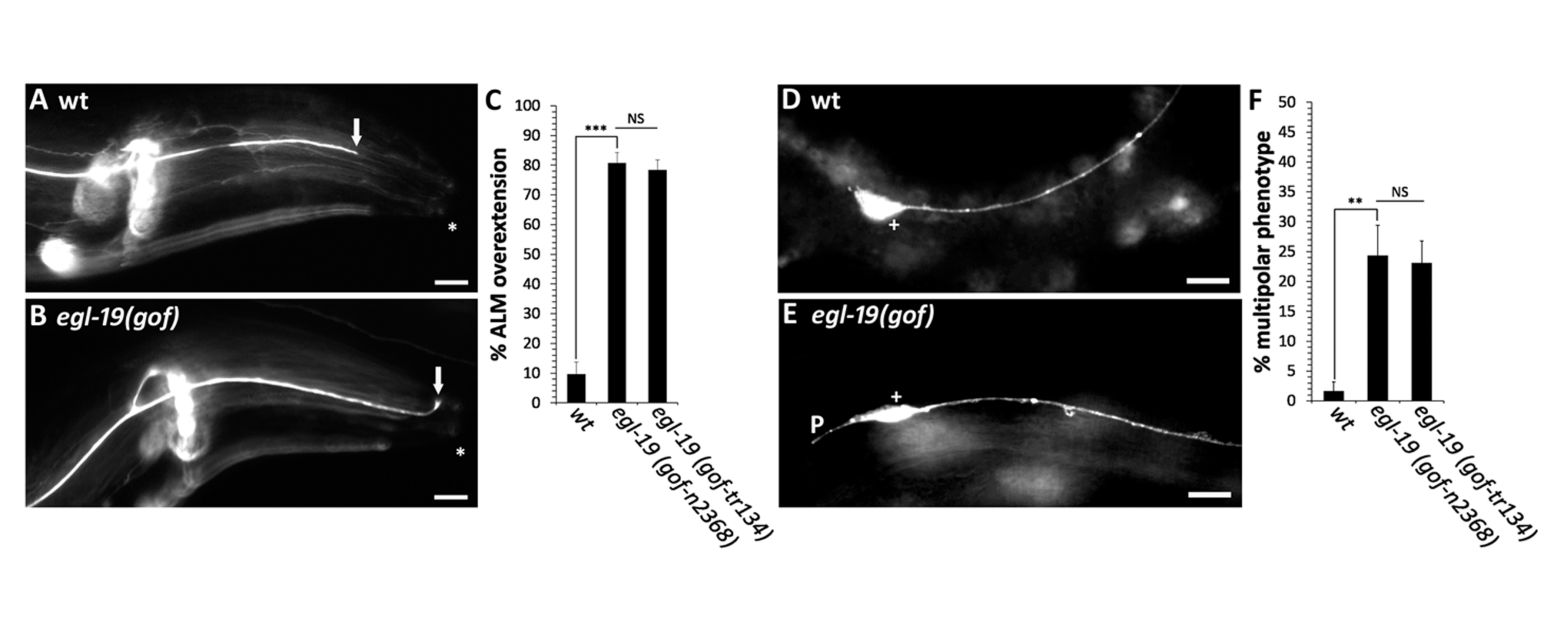
(A) Example of normal axon termination in a wild-type ALM neuron, where the axon terminates posterior to the tip of the nose (arrow). (B) Example of axon termination defect in *egl-19(gof)* mutants, where the ALM axon extends to the tip of the nose (arrow). (C) Quantification of axon overextension defects in ALM neurons. (D) Example of a normal cell body of a wild-type ALM neuron, where a single process extends from the anterior side of the ALM cell body. (E) Example of a multipolar phenotype in *egl-19(gof)* mutants, where a short process extends from the posterior side of the ALM cell body. (F) Quantification of the multipolar phenotype that is caused by the *egl-19(gof)* mutants. Axons are visualized with the *muIs32* transgene that encodes *Pmec-7::gfp*. Arrows point to the tip of the ALM axon. Asterisk marks the anterior-most part of the worm. + indicates ALM cell body. P indicates a multipolar defect. Scales bars are 10um. Between 100 and 150 axons were observed in L4 stage hermaphrodites per genotype. Asterisks indicate statistically significant difference, Z-test for proportions (**p<0.0005; ***p<0.0001). Error bars represent the standard error of the proportion.

## Description

The *egl-19* gene in *C. elegans* encodes the pore forming subunit for the L-type voltage gated calcium channel that is homologous to the *CACNA1C* gene in humans (Lee *et al.*, 1997). Variants in *CACNA1C* are risk factors for autism and other neurodevelopmental disorders (Li *et al.*, 2015; Lu *et al.*, 2012; Strom, *et al.*, 2010). Timothy syndrome is a syndromic form of autism that can be caused by either of three rare *de novo* mutations in *CACNA1C.* These mutations cause either a G402R, G402S or G406R mutation in the CACNA1C protein (Splawski *et al.*, 2004; Bader *et al.*, 2011). Our previous work demonstrated that PLM axon termination is disrupted by mutations equivalent to the G402R and G406R mutations in *CACNA1C* (Buddell *et al.*, 2019). Our study also revealed behavioral defects in these mutant worms. Although the anatomical basis for these behavioral defects has not been determined, it is likely that they are caused by multiple defects within the mechanosensory system.

To learn more about how the Timothy syndrome mutations can alter neuronal development, we focused on the *egl-19(n2368)* and *egl-19(tr134)* mutations (Lee *et al.*, 1997; Kwok *et al.*, 2008), hereafter called the *egl-19(gof)* mutations*.* Both of these *egl-19(gof)* mutations lead to a G365R amino acid change in EGL-19 that is equivalent to the G402R gain of function mutation in *CACNA1C* that can cause Timothy syndrome in humans. Here, we report that the *egl-19(gof)* mutations can cause the growth of an ectopic process from the cell body of the ALM neuron. This observation suggests that in addition to causing defects in axon termination, the *egl-19(gof)* mutations can also disrupt the polarity of process outgrowth.

The mechanosensory neurons in *C. elegans* are responsible for transducing light touch and consist of two ALM neurons, two PLM neurons, one AVM neuron and one PVM neuron (Chalfie *et al.*, 1985). To identify neuronal defects caused by the *egl-19(gof)* mutations, we labeled each of the six mechanosensory neurons with a fluorescent transgene that is expressed in each of the six mechanosensory neurons. After observing each of the six mechanosensory neurons in populations of *egl-19(gof)* mutants, we identified a novel phenotype in the ALM neuron. In wild-type animals, nearly all ALM neurons extend a single process from the anterior side of the cell body (Figure 1D,F). However, in *egl-19(gof)* animals, we often observed a second short process that extended in the posterior direction (Figure 1E,F). In addition to this novel phenotype, we also found an axon termination defect in ALM neurons that is similar to the axon termination defect that we previously reported in the PLM neuron. In wild-type animals, the cell bodies of the ALM neurons reside on the lateral body wall and extend a single axon into the head, where they terminate prior to reaching the tip of the nose (Figure 1A). In *egl-19(gof)* mutants, we observed overextended ALM axons, where the axon extended past its normal termination point and terminated within the tip of the nose (Figure 1B,C).

These results suggest that the Timothy syndrome mutation can disrupt multiple steps of axon development. First, the *egl-19(gof)* mutations can disrupt the polarization of process outgrowth. Second, the *egl-19(gof)* mutations can also disrupt axon termination. Future work will address the molecular mechanisms that underlie these alterations in neuronal polarity and axon termination. An understanding of these mechanisms will be critical to our understanding of how variants in *CACNA1C* give rise to the axonal defects that underlie autism.

## Methods

*C. elegans* strains were cultured and maintained on nematode growth medium (NGM)-agar plates using standard methods at 20°C (Brenner, 1974). Axons were labeled and observed as previously described (Xu *et al.*, 2012). Briefly, animals were mounted on a 5% agarose pad and observed with a 40x objective. PLM & ALM neurons were visualized with the *muIs32* transgene which encodes *Pmec-7::gfp* + *lin-15(+)* and is expressed in all mechanosensory neurons (Ch’ng *et al.*, 2003). The microscope used for imaging and phenotype analysis was the Zeiss Axio Imager M2. Images were acquired using an AxioCam MRm camera and were analyzed using Axiovision 4 software.

## Reagents

AGC48: *muIs32* [*mec-7*p::GFP + *lin-15*(+)]II; *egl-19(n2368)* IV

AGC139: *muIs32* [*mec-7*p::GFP + *lin-15*(+)]II; *egl-19(tr134)* IV
